# Mental Disorders and Quality of Life in Patients With Pulmonary Arterial Hypertension Treated With Sotatercept

**DOI:** 10.1002/pul2.70324

**Published:** 2026-05-21

**Authors:** Jan Fuge, Karen M. Olsson, Kira Remmert, Britta Stapel, Jan C. Kamp, Da‐Hee Park, Marius M. Hoeper, Lotta Winter, Kai G. Kahl

**Affiliations:** ^1^ Department of Respiratory Medicine and Infectious Diseases Hannover Medical School Hannover Germany; ^2^ Biomedical Research in Endstage and Obstructive Lung Disease Hannover (BREATH), German Center for Lung Research (DZL) Hannover Germany; ^3^ Department of Psychiatry, Social Psychiatry and Psychotherapy Hannover Medical School Hannover Germany

**Keywords:** metacognition, patient‐reported outcomes, psychosocial adaptation, structured clinical interview for DSM‐5 (SCID)

## Abstract

Sotatercept has recently expanded treatment options for pulmonary arterial hypertension (PAH), but its mental health and quality‐of‐life impact is not well characterized. We assessed psychiatric diagnoses and patient‐reported outcomes in PAH patients treated with sotatercept within the clinical trial program. PAH Patients previously assessed for psychiatric disorders and quality of life were re‐evaluated after ≥ 6 months of sotatercept exposure. A senior psychiatrist performed a Structured Clinical Interview for DSM‐5, and participants completed validated questionnaires assessing anxiety/depression (HADS), metacognitive beliefs (MCQ‐30), quality of life (WHOQOL‐BREF), and PAH‐related symptoms/impacts (PAH‐SYMPACT™), alongside items capturing patient experiences during therapy. Twenty patients (85% female; median age 46 years) were included. Hemodynamics, biomarkers, and 6‐min walk distance improved. The prevalence of at least one current psychiatric disorder remained high and largely unchanged from baseline to follow‐up (50% to 45%). Major depressive disorder remained stable (25%), whereas adjustment disorder newly occurred in 25% of patients. HADS and MCQ‐30 scores did not change. WHOQOL‐BREF global scores increased modestly, but the mental health domain declined substantially (69.6 ± 13.3 to 48.1 ± 15.5, *p* = 0.001). PAH‐SYMPACT™ indicated low symptom burden at follow‐up. Many patients reported increased optimism and plans to travel or return to work, while some expressed concerns about finances and social security. While sotatercept treatment led to clinical improvements in PAH patients, the prevalence of mental disorders remained high with an increased prevalence in adjustment disorder. These findings support routine mental health assessment and targeted psychosocial support when initiating life‐changing PAH therapies.

## 1 Introduction

Pulmonary arterial hypertension (PAH), a life‐threatening condition, is characterized by vascular remodeling of the pulmonary arteries resulting in increased pressure, resistance and right ventricular failure if not adequately treated [1]. Existing treatments offer limited survival benefits, necessitating novel approaches [2, 3].

The diagnosis of pulmonary arterial hypertension (PAH) elicits complex emotions in patients, encompassing relief at finding an explanation for symptoms and the realization of a severe, permanent, and potentially life‐threatening condition. This diagnosis brings profound consequences, including coping with physical limitations, uncertainties about the future, potential work‐related challenges with financial and social repercussions, concerns about family planning, the necessity of ongoing medical check‐ups and invasive procedures, and the complexities and side effects of medical therapies, including the potential need for lung transplantation [4, 5, 6].

In a previous study, Olsson et al. systematically assessed psychiatric disorders in PAH patients through structured face‐to‐face interviews, revealing several key findings: Over one‐third of patients had psychiatric disorders, predominantly major depressive disorder and panic disorder, exceeding general population prevalence [6]. Psychiatric disorders significantly impacted patients' quality of life (QoL), with major depressive disorder associated with a 13% decline and panic disorder with an 8% decline in WHOQOL‐BREF‐Score. Additionally, signs of adjustment disorder within the first 3 months post‐PAH diagnosis were reported by more than one‐third of patients, with over half developing other psychiatric disorders subsequently [6].

Sotatercept, an activin signaling inhibitor, has recently been proven effective in several independent clinical trials, showing robust improvements in pulmonary hemodynamics, exercise capacity, QoL, and outcomes [7, 8, 9, 10]. The impact of sotatercept on mental disorders remains largely unexplored. Furthermore, clinical observation indicated that despite or even due to clinical improvement and the change of perspectives, other psychosocial burdens arose for the patients. This study seeks to investigate potential alterations in mental health, metacognitive beliefs, and QoL among patients undergoing sotatercept treatment.

## 2 Methods

### 2.1 Patient Setting and Clinical Parameters

**Figure 1:**
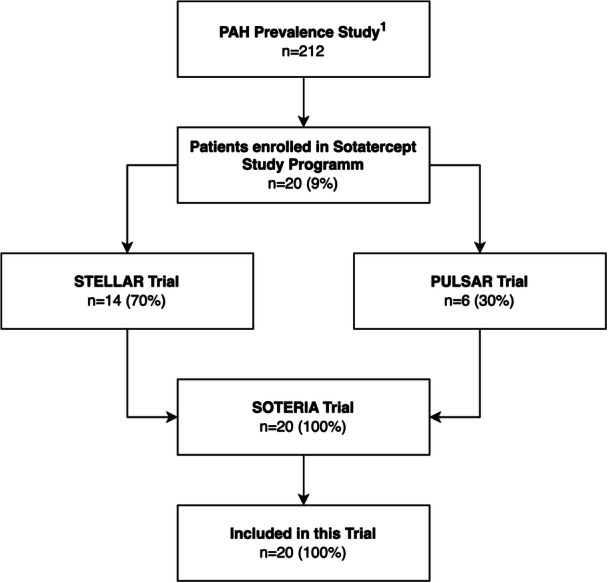
Study Flowchart, ^1^Olsson et al. 2021.

We performed a longitudinal within‐subject re‐assessment of psychiatric diagnoses and quality of life in patients with pulmonary arterial hypertension (PAH) who had previously undergone a Structured Clinical Interview for DSM‐5 (SCID) in our prior study [6]. Participants were eligible if they (i) had taken part in that earlier assessment of mental disorders and quality of life and (ii) subsequently received sotatercept for at least 6 months within the sotatercept clinical trial program. This program comprised the PULSAR, STELLAR, and ZENITH trials and their open‐label extension study, SOTERIA [8, 9, 10]. All participants were ≥ 18 years of age and able to complete questionnaires and interviews in German. The study flow is shown in Figure 1. The institutional review board approved the study (No. 8450_BO_K_2019), and all patients provided written informed consent.

### 2.2 Clinical Data

Clinical data were collected immediately before treatment with sotatercept and after at least 6 months of sotatercept treatment. Clinical assessments encompassed age, height, weight, body mass index (BMI) and smoking status, 6‐min walking distance (6MWD), World Health Organization functional class (WHO‐FC), and serum levels of the N‐terminal fragment of proBNP (NT‐proBNP). Hemodynamic data from right heart catheterization prior sotatercept initiation and after at least 6 months on therapy were recorded. Additionally, sociodemographic information, including educational and employment status were obtained through self‐rated questionnaires.

### 2.3 Assessment of Psychometric Status, Mental Disorders and Quality of Life

Patients answered an online questionnaire using SoSciSurvey online questionnaire version 3.5.04 (SoSci Survey GmbH, Munich, Germany) comprising questions regarding QoL, mental disorders, metacognitive beliefs, and symptoms of anxiety and depression.

QoL was assessed using the WHOQOL‐BREF [11], a short form, 30 items questionnaire resulting in five domains global, physical, mental, social and environmental. Scoring results in points between 0 (worst) and 100 (best) quality of life per domain. Additionally, patients completed the PAH‐SYMPACT™, a 23‐item Patient‐Reported Outcome (PRO) tool for assessing symptoms and impacts of PAH, which was also used in the STELLAR‐trial. Symptom items were divided into cardiopulmonary and cardiovascular domains, while impact items are categorized into physical and emotional/cognitive domains. Domain scores ranging from 0, no symptom at all to 4, very severe symptom, were calculated average item score within each domain.

Symptoms of anxiety and depression were assessed using the Hospital Anxiety and Depression Scale (HADS) [12] containing seven questions for assessment of anxiety and depression, respectively, with a 4‐point Likert‐scale (0‐3 points) resulting in a score between 0 and 21 points. Scores from 0 to 7 indicate no symptoms of anxiety or depression, from 8 to 10 points probable symptoms and ≥ 11 points definite symptoms. Metacognitive beliefs were assessed using the MCQ‐30 [13], a short form 30 items questionnaire with a 4 point Likert‐scale (1‐4 points) resulting in five subdomains: positive beliefs about worry, negative beliefs about thoughts concerning uncontrollability and danger, cognitive confidence, negative beliefs concerning the consequences of not controlling thoughts and cognitive self‐consciousness. A higher score is associated with more dysfunctional beliefs.

### 2.4 Sotatercept Related Questions

Given the clinical and hemodynamic improvements under sotatercept therapy, a list of statements/questions were compiled covering various areas derived from clinical observations and patient inquiries documented in the clinical database. These statements addressed potential new problems arising when clinical symptoms disappear, including social security, work capability, travel desires, family planning, and feelings of guilt for benefiting from therapy when others did not. A five‐point Likert scale (1 = strongly disagree to 5 = strongly agree) was used for responses, and patients were given the opportunity to provide additional free text comments on how sotatercept treatment and related physical consequences affected their lives.

### 2.5 Structured Clinical Interview for DSM‐5 (SCID) for Psychiatric Diagnosis

In addition to the questionnaires, a senior psychiatrist conducted a structured clinical interview for DSM‐5 (SCID) [14], either in person or via phone call. Mental disorder diagnoses were determined according to the Diagnostic and Statistical Manual of Mental Disorders, Fifth Edition (DSM‐5).

### 2.6 Statistical Analysis

IBM SPSS Statistics 29.0 (IBM Corp, Armonk, NY, USA) and R environment for statistical computing version 4.1.2 (R Foundation for Statistical Computing, Vienna, Austria) were used for data analysis and figure creation. Continuous variables are shown as median and interquartile range (Q_25_ to Q_75_, IQR) or as mean and standard deviation (SD) depending on distribution, as appropriate. Categorical variables are shown as number and percent (%), unless indicated otherwise. Wilcoxon signed‐rank test or McNemar‐test was used to compare parameters prior sotatercept and after at least 6 months on sotatercept. Exploratory subgroup analysis for psychometric parameters and QoL by (i) baseline prostacyclin analogue therapy (PCA) and (ii) improvement of WHO FC, were conducted. Further, comparisons of prevalence of mental disorders were conducted based on the previous study by Olsson et al. [6]. A *p*‐value of 0.05 was considered significant.

## 3 Results

A total of *n* = 20 patients (85% female, median age 46 years (interquartile range, 39–56)) were enrolled of which 14 (70%) originated from the STELLAR trial and 6 (30%) from the PULSAR trial (Table 1 and Figure 1). Within the first 6 months of treatment with sotatercept, these patients experienced improvements in hemodynamics, right heart function and exercise capacity as shown in Table 2.

**Table 1:** Demographics and clinical characteristics after 6 months on sotatercept.

	Patient cohort *n* = 20
Age (years)	46 (39–56)
Female sex (%)	17 (85%)
BMI (kg/m²)	26.1 (21.8–31.3)
Diagnosis	
IPAH, *n* (%)	14 (70%)
HPAH, *n* (%)	5 (25%)
APAH, *n* (%)	1 (5%)
Time since diagnosis (years)	10 (7–13)
PAH medication prior sotatercept	
Double oral combination therapy, *n* (%)	4 (20%)
Triple oral combination therapy, *n* (%)	4 (20%)
Triple combination therapy with iv/sc PCA, *n* (%)	12 (60%)

*Note:* Continuous variables are stated as median and interquartile ranges (IQR) and categorical variables are stated as n and percent (%), unless indicated otherwise.

Abbreviations: BMI, body mass index; I/H/APAH, idiopathic, heritable or associated pulmonary arterial hypertension; iv/sc, intravenous, subcutaneous; IQR, interquartile range; PCA, prostacyclin analogues; WHO FC, World Health Organization Functional Class.

**Table 2:** Clinical parameters before sotatercept and after at least 6 months on sotatercept treatment.

	Before sotatercept *n* = 20	6 months on sotatercept *n* = 20	*p*‐value
Hemodynamics			
mPAP (mmHg)	57 (51–67)	52 (30–55)	**0.046**
PAWP (mmHg)	9 (7–11)	7 (6–10)	0.276
CI (L/min/m²)	2.5 (2.2–2.8)	2.6 (2.0–3.6)	0.734
PVR (dyn*s*cm‐5)	899 (655–1034)	599 (418–762)	0.176
WHO FC			
I/II, *n* (%)	7 (65%)	13 (65%)	0.070
III, *n* (%)	13 (65%)	7 (35%)	
IV, *n* (%)	0 (0%)	0 (0%)	
6MWD (m)	437 (404–453)	453 (424–491)	**< 0.001**
NT‐proBNP (ng/L)	704 (289–2045)	155 (76–657)	**< 0.001**

*Note:* Continuous variables are stated as median and interquartile ranges (IQR) and categorical variables are stated as *n* and percent (%), unless indicated otherwise. *p*‐values were calculated using paired Wilcoxon signed‐rank test or McNemar‐test. Statistically significant values in bold.

Abbreviations: BNP, brain natriuretic peptide; CI, cardiac index; IQR, interquartile range; mPAP, mean pulmonary arterial pressure; NT‐proBNP, N‐terminal fragment of pro‐brain natriuretic peptide; PAH, pulmonal arterial hypertension; PAWP, pulmonary artery wedge pressure; PVR, pulmonary vascular resistance; WHO FC, World Health Organization Functional Class; 6MWD, 6‐min walking distance.

### 3.1 Prevalence of Mental Disorders

At baseline, 10/20 patients (50%) met SCID criteria for at least one current mental disorder, most commonly MDD (25%) and specific phobia (10%); adjustment disorder was absent. After 6 months on sotatercept, overall prevalence was similar (9/20, 45%). MDD (25%) and agoraphobia (5%) were unchanged, specific phobia declined to 5%, but five new cases of adjustment disorder emerged (25%). All changes non‐significant (Table 3).

**Table 3:** Prevalence of common mental disorders at timepoint 1 (prior sotatercept) compared to timepoint 2 (after at least 6 months of sotatercept treatment).

	Before sotatercept *n* = 20	6 months on sotatercept *n* = 20	*p*‐value
Any current mental disorder	50% (29.9–70.1)	45% (25.8–65.8)	1.000
Alcohol abuse	0 (0)	0 (0)	—^a^
Alcohol dependence	0 (0)	0 (0)	—^a^
Schizophrenia	0 (0)	0 (0)	—^a^
Major depressive disorder	25% (11.2–46.9)	25% (11.2–46.9)	1.000
Bipolar 1 disorder	0 (0)	0 (0)	—^a^
Panic disorder	0 (0)	0 (0)	—^a^
Agoraphobia	5% (0.9–23.6)	5% (0.9–23.6)	1.000
Social phobia	0 (0)	0 (0)	—^a^
Adjustment disorder	0 (0)	25% (11.2–46.9)	—^a^
Specific phobia	10% (2.8–30.1)	5% (0.9–23.6)	1.000
PTSD	0 (0)	0 (0)	—^a^
Obsessive compulsive disorder	5% (0.9–23.6)	0 (0)	—^a^
Anorexia nervosa	0 (0)	0 (0)	—^a^
Bulimia nervosa	0 (0)	0 (0)	—^a^

*Note:* Psychological disorder prevalence rates per group stated as percent (%) and 95% CIs. Statistically significant values in bold. ^a^Samples do not satisfy the standard binomial requirement.

Abbreviations: PAH, pulmonary arterial hypertension; PTSD, posttraumatic stress disorder.

### 3.2 Patient‑Reported Anxiety, Depression and Metacognitions

Hospital Anxiety and Depression Scale scores remained unchanged (HADS‑A 5.2  ± 4.7 vs 5.5  ±  3.7, *p* = 0.943; HADS‑D 5.0  ± 4.6 vs 5.1  ± 3.8, *p* = 0.431). MCQ‑30 total and sub‑scale scores showed no significant differences (Table 4).

**Table 4:** QoL and psychometric scores in PAH patients before sotatercept compared to at least 6 months on sotatercept treatment.

Scores in points	Before sotatercept *n* = 20	6 months on sotatercept *n* = 20	*p*‐value
WHOQOL‐BREF			
Global	53.8 ± 16.8	60.2 ± 18.0	0.786
Physical	60.2 ± 13.3	56.2 ± 16.5	0.058
Mental	69.6 ± 13.3	48.1 ± 15.5	**0.001**
Social	62.9 ± 21.2	56.2 ± 18.4	0.313
Environmental	78.0 ± 13.4	76.2 ± 12.7	0.295
PAH‐SYMPACT^TM^			
Cardio‐pulmonal symptoms	—	1.2 ± 0.6	—
Cardiovascular symptoms	—	0.4 ± 0.3	—
Physical impact	—	1.3 ± 0.9	—
Cognitive impact	—	1.3 ± 0.9	—
HADS			
Total score	10.2 ± 8.4	10.6 ± 6.6	0.517
HADS‐A Score	5.2 ± 4.7	5.5 ± 3.7	0.943
HADS‐D Score	5.0 ± 4.6	5.1 ± 3.8	0.431
MCQ‐30			
Total score	45.4 ± 11.6	48.8 ± 12.0	0.437
Positive beliefs about worry	7.5 ± 1.8	7.8 ± 2.5	0.217
Negative beliefs	9.4 ± 4.0	10.4 ± 3.9	0.326
Cognitive confidence	9.5 ± 3.2	10.3 ± 3.3	0.565
Need to control thoughts	9.1 ± 3.1	9.4 ± 3.2	0.378
Cognitive self‐consciousness	9.9 ± 3.3	10.8 ± 3.6	0.597

*Note:* Continuous variables are stated as mean and standard deviation (SD) and categorical variables are stated as *n* and percent (%), unless indicated otherwise. *p*‐values were calculated using paired Wilcoxon signed‐rank test. Statistically significant values in bold.

Abbreviations: A, anxiety; D, depression; HADS, hospital anxiety and depression scale; MCQ‐30, Metacognitions questionnaire 30; QoL, quality of life; WHO, world health organization.

### 3.3 Quality of Life

Global WHOQOL‑BREF scores rose modestly without statistical significance (53.8  ± 16.8 to 60.2  ±  18.0, *p* = 0.786). Physical, social and environmental domains were stable, whereas the mental domain declined markedly from 69.6  ± 13.3 to 48.1  ± 15.5 (*p* = 0.001). PAH‐SYMPACT™ revealed minimal symptoms in the cardiopulmonary domain, as well as low levels of physical and cognitive impact following sotatercept treatment, with scores of 1.2 ± 0.6, 1.3 ± 0.9, and 1.3 ± 0.9, respectively. Remarkably low scores were observed in the cardiovascular symptom domain, with a score of 0.4 ± 0.3. However, baseline values were not available for comparison (Table 4).

Exploratory subgroup analyses showed that the decline in the WHOQOL‐BREF mental domain was not confined to patients without WHO functional class improvement, but was also observed in those with improved WHO functional class: mental‐domain scores decreased from 69.4 ± 22.4 to 44.8 ± 17.5 in patients without improvement (*p* = 0.008) and from 69.8 ± 16.8 to 53.1 ± 13.7 in those with improvement (*p* = 0.039), see Tables S1 and S2).

Regarding the specific questions related to the impact of sotatercept treatment, 9 patients (53%) reported a more positive outlook on life, and 10 patients (59%) expressed hopes for a normal life expectancy for the first time since diagnosis. Ten patients (59%) were planning to resume traveling, while six patients (35%) had taken up new hobbies. Additionally, seven patients (41%) indicated a return to their previous jobs. Concerns about losing social security benefits were expressed by four patients (24%), and five patients (29%) felt anxious about their economic well‐being; one patient (6%) had no pension entitlement.

Changes in relationships with partners were reported by three patients (18%), two of which were reported to be associated with sotatercept‐associated changes in life perspectives. One patient (6%) expressed a heightened desire to have children compared to before the study. Furthermore, one patient (6%) reported feeling guilty that others were doing worse without study medication, 1 (6%) felt guilty about other patients' deaths, and 2 (12%) felt guilty that some patients could not participate in the study. Additional details are provided in Table S3.

Nine patients receiving sotatercept highlighted various aspects relevant to their lives in free text format, covering the following topics: Patients described significant psychological challenges arising from the fluctuating nature of side effects during the study, aggravated by personal circumstances. Despite experiencing an improvement in their condition compared to before sotatercept treatment, some patients reported dizziness and recurrent tachycardia, leading to doubts about the stability of their health. Some expressed hopes that increased activity would facilitate social engagement, while others harbored concerns about potential relapses undermining the improvements gained from the study.

There was a prevailing sense of cautious optimism about the study's outcomes, coupled with a fear of future setbacks. Patients emphasized the importance of ongoing research for further advancements and, ideally, a cure, though they acknowledged the lengthy process involved. Coping with the challenges posed by treatment, such as finding the courage to push physical limits and managing the fear of relapse, was a common theme among patients. Additionally, there was a pervasive fear of treatment failure and uncertainty about life expectancy.

Patients also highlighted societal misconceptions surrounding their illness, often feeling misunderstood or underestimated. Reflecting on the impact of their illness, many expressed regrets over missed opportunities and lost time over the years. Moreover, patients faced the challenge of adapting to a new physical sensation, identifying personal boundaries, and grappling with identity shifts due to their condition.

## 4 Discussion

The primary findings of this study can be summarized as follows: (i) despite clinical improvement, the prevalence of mental disorders persisted in patients with PAH treated with sotatercept; the prevalence of adjustment disorder increased in our cohort, (ii) metacognitive beliefs remained unchanged and (iii) the availability of sotatercept affected patients' outlook, hopes, and lifestyle, indicating multifaceted impact on both physical and psychosocial aspects. These findings suggest that the introduction of sotatercept therapy presents new challenges for both patients and healthcare professionals.

The findings of this study provide valuable insights into the multiple effects of sotatercept treatment in patients with PAH. The enrolled cohort, consisting predominantly of young females, exhibited a high prevalence of mental disorders at baseline, particularly MDD and increasing adjustment disorder. Adjustment disorder is a psychological condition characterized by an emotional or behavioral response to a stressful life event of change that is considered maladaptive compared to what would normally be expected. This reaction typically occurs within 3 months of the identified stressor and causes significant psychosocial impairment in daily life. The condition frequently resolves within 6 months. Importantly, adjustment disorder may arise not only after clinical deterioration, but also during major transitions in illness trajectory, when patients must adapt to new expectations, regained options, and uncertainty about whether improvement will last. In this context, rapid therapeutic change may itself function as a psychosocial stressor by challenging established roles, coping strategies, and illness identity. Similar adaptation difficulties have been described in other chronic disease settings after major therapeutic advances, including cystic fibrosis after highly effective CFTR modulator therapy and cancer survivorship after successful treatment [15, 16]. Patients in our study exhibited mostly emotional distress. Some patients reported fear of recurrence of symptoms, some a diminishing effect of the treatment. This underscores the importance of considering mental health comorbidities in PAH management, as they can significantly influence patient well‐being and treatment outcomes. Systematic re‐assessment at 9–12 months (and beyond) is needed to determine whether the burden of adjustment disorder decreases once patients have had more time to consolidate their ‘new normal’.

In terms of patient‐reported outcomes, patients reported minimal symptoms in the cardiopulmonary domain and low levels of physical and cognitive impact, as measured by PAH‐SYMPACT™. Yet QoL findings were more nuanced: while the WHOQOL‑BREF global score rose modestly without reaching statistical significance, and other domains remained stable, the mental‑health domain deteriorated markedly. This divergence ‐ stable HADS scores but a pronounced drop in perceived mental well‑being ‐ highlights the need for targeted psychosocial support alongside somatic treatment gains. Similar findings have been observed in other chronic conditions benefiting from pharmacological breakthroughs. For example, patients with cystic fibrosis treated with highly effective CFTR modulator therapy reported transformative physical improvements but continued to experience psychological distress, identity changes, and adjustment challenges. This led to calls for proactive psychological support to help patients adapt to their ‘new normal’ [15]. Remarkably, patients reported positive changes in outlook and aspirations for the future following initiation of sotatercept treatment. For the first time since receiving the PAH diagnosis, patients expressed hopes for the prospect of a normal life expectancy. Moreover, the desire to resume activities such as traveling, engaging in new hobbies and starting or going back to work highlights the importance of treatment efficacy. These aspirations underline that therapeutic success should be judged not only by hemodynamic and functional improvement but also by the extent to which patients can reclaim meaningful roles and social participation. In some patients, the marked and unexpected improvement with sotatercept created a new kind of crises as they started to become less dependent on their caregivers, sometimes leading to a break‐up of long‐standing partnerships. Some patients became afraid of losing their social coverage as they became fit enough to start or go back to work. Particularly in patients who had been unable to work during their entire adult life due to PAH, this occasionally created fears and anxieties.

In addition, patients voiced concerns about a potential loss of treatment efficacy over time, potential relapses, and uncertainties regarding life expectancy. New side effects of pre‐existing therapies aggravated by sotatercept as well as side‐effects linked to sotatercept itself may have contributed to those uncertainties as they might not be easy to discriminate from clinical worsening at first. These concerns might have contributed to the reduced QoL, especially in the mental domain, underscoring the need for continued monitoring and support to address patient anxieties and ensure ongoing adherence to established but also new and potentially life‐saving treatment regimens. Such fears are not unique to PAH; cancer survivorship research has shown that fear of disease recurrence is highly prevalent and strongly associated with reduced quality of life, even in patients in remission [16].

Patients also highlighted societal misconceptions and challenges in adapting to their illness, emphasizing the importance of holistic care approaches that address not only physical symptoms but also psychosocial and identity‐related concerns. Healthcare providers must strive to validate patients' experiences and provide tailored support to help them navigate the complexities of living with PAH and undergoing sotatercept therapy.

In practice, support programs around major treatment transitions may combine routine mental‐health screening with structured referral pathways, psychoeducation, and peer or self‐management support. In cystic fibrosis, international mental‐health guidelines have successfully embedded annual depression/anxiety screening and treatment pathways into routine specialty care, providing a useful model for rare chronic diseases with complex treatment trajectories [17]. In addition, a randomized clinical trial in patients with rare chronic diseases showed that a brief peer‐delivered self‐management intervention improved disease acceptance [18], while a survey in pulmonary hypertension suggested that support‐group participation may improve meaningful health‐related outcomes even when global quality‐of‐life scores remain unchanged [19]. Together, these data support integrating psychological screening, social counseling, and peer‐based support into PH programs when patients initiate life‐changing therapies.

## 5 Limitations

This study is limited by its low number of patients, monocentric design, and lack of a control group. Further, we only captured one post‐baseline time‐point (6 months); thus, we could not map the full temporal arc of adjustment disorders. However, little data on the psychometric impact of sotatercept treatment has been published so far, and our findings indicate that the psychosocial consequences of introducing life‐changing therapies are more complex than anticipated. In addition, the hemodynamic and functional improvements of the patients in our cohort were relatively small compared to the overall response reported with sotatercept, potentially limiting the generalizability of our findings.

## 6 Conclusion

In conclusion, while sotatercept shows potential positive effects on various aspects of patients' lives, including mental health and quality of life, the study underlines the multifaceted challenges faced by individuals with PAH. While showing modest differences in the occurrence of mental disorders, patient treated with sotatercept exhibited a decline in mental quality of life and an improvement in positive beliefs about worry. Despite these challenges, patients reported positive outlooks on life, aspirations for normalcy, and various lifestyle changes. The study emphasizes the need for a deeper understanding of the intricate interplay between PAH, its treatment, and the complex psychosocial aspects faced by patients. PH programs adopting sotatercept should consider proactive mental health screening and counseling around work/disability and life planning.

## Author Contributions

**Jan Fuge:** conceptualization, methodology, investigation, resources, formal analysis, visualization, writing – original draft. **Karen M. Olsson:** conceptualization, methodology, investigation, resources, supervision, writing – review and editing. **Kira Remmert:** investigation, resources, writing – review and editing. **Britta Stapel:** investigation, writing – review and editing. **Jan C. Kamp:** investigation, writing – review and editing. **Da‐Hee Park:** investigation, writing – review and editing. **Marius M. Hoeper:** methodology, investigation, resources, writing – original draft. **Lotta Winter:** investigation, writing – original draft. **Kai G. Kahl:** conceptualization, methodology, investigation, resources, formal analysis, writing – original draft.

## Ethics Statement

The institutional review board approved the study (No. 8450_BO_K_2019), and all patients provided written informed consent.

## Conflicts of Interest

Jan Fuge has nothing to disclose. Karen M Olsson has received fees for lectures and/or consultations from Acceleron, Actelion, AOP Health, Janssen, Ferrer, Merck, AOP Health, Gossamer, Bayer and OMT; all outside the present work. Kira Remmert has nothing to disclose. Britta Stapel has nothing to disclose. Jan C Kamp has nothing to disclose. Da‐Hee Park has nothing to disclose. Marius M Hoeper has received fees for consultations or lectures from 35Pharma, Acceleron, Actelion, Aerovate, AOP Health, Bayer, Ferrer, Gossamer, Inhibikase, Janssen, Keros, MSD and Novartis. Lotta Winter has nothing to disclose. Kai G Kahl has nothing to disclose.

## Supporting information


**Table S1:** QoL and psychometric scores in PAH patients before sotatercept compared to at least 6 months on sotatercept treatment by Baseline PCA therapy. **Table S2:** QoL and psychometric scores in PAH patients before sotatercept compared to at least 6 months on sotatercept treatment by change of WHO FC. **Table S3:** Sotatercept specific questions after at least 6 months on sotatercept treatment – n=17 patients participated.

## Data Availability

The data that support the findings of this study are available on request from the corresponding author. The data are not publicly available due to privacy or ethical restrictions. Data of this research can be obtained by the corresponding author on reasonable request.
