# Supplementary material for: Mental Disorders and Quality of Life in Patients With Pulmonary Arterial Hypertension Treated With Sotatercept
Source: Pulm Circ. 2026 May 21;16(2):e70324. doi: 10.1002/pul2.70324 (PMC13239444; doi:10.1002/pul2.70324)
Supplement: Supplementary file 1 — Table S1: QoL and psychometric scores in PAH patients before sotatercept compared to at least 6 months on sotatercept treatment by Baseline PCA therapy. Table S2: QoL and psychometric scores in PAH patients before sotatercept compared to at least 6 months on sotatercept treatment by change of WHO FC. Table S3: Sotatercept specific questions after at least 6 months on sotatercept treatment – n=17 patients participated. [file PUL2-16-e70324-s001.docx]

**Table S1 - QoL and psychometric scores in PAH patients before sotatercept compared to at least 6 months on sotatercept treatment by Baseline PCA therapy**

|  | Without PCA (n=8) | | | With PCA (n=12) | | |
| --- | --- | --- | --- | --- | --- | --- |
| Scores in points | **Before sotatercept** | **6 months on sotatercept** | **p value** | **Before sotatercept** | **6 months on sotatercept** | **p value** |
| WHOQOL-BREF |  |  |  |  |  |  |
| - Global | 50.0 ±16.4 | 54.2 ±15.1 | 0.850 | 56.2 ±17.3 | 58.0 ±23.2 | 0.932 |
| - Physical | 58.5 ±11.0 | 48.2 ±14.1 | 0.141 | 61.3 ±15.0 | 56.2 ±15.2 | 0.238 |
| - Mental | 65.1 ±15.6 | 48.8 ±9.8 | 0.219 | 72.6 ±22.4 | 48.7 ±18.9 | **0.002** |
| - Social | 59.4 ±18.6 | 72.2 ±15.5 | 0.272 | 65.3 ±23.3 | 52.3 ±15.9 | **0.011** |
| - Environmental | 72.7 ±10.3 | 81.8 ±11.6 | 0.520 | 81.5 ±14.4 | 72.4 ±14.3 | **0.025** |
| HADS |  |  |  |  |  |  |
| Total score | 13.0 ±7.6 | 9.3 ±4.9 | 0.598 | 8.2 ±8.6 | 11.3 ±7.6 | 0.101 |
| HADS-A Score | 7.0 ±4.9 | 5.3 ±4.0 | 0.351 | 3.9 ±4.4 | 5.6 ±3.6 | 0.163 |
| HADS-D Score | 6.0 ±5.0 | 4.0 ±2.1 | 0.611 | 4.3 ±4.4 | 5.8 ±4.8 | 0.118 |
| MCQ-30 |  |  |  |  |  |  |
| - Total score | 50.5 ±12.5 | 52.3 ±14.3 | 1.000 | 41.9 ±10.1 | 45.8 ±13.0 | 0.506 |
| - Positive beliefs about worry | 8.0 ±2.5 | 7.7 ±2.4 | 0.581 | 7.1 ±1.1 | 6.7 ±1.8 | 0.268 |
| - Cognitive confidence | 10.4 ±3.0 | 11.3 ±3.1 | 0.588 | 8.9 ±3.2 | 9.6 ±3.0 | 0.343 |
| - Negative beliefs | 10.5 ±3.5 | 10.8 ±4.3 | 1.000 | 8.7 ±4.3 | 10.7 ±4.9 | 0.283 |
| - Need to Control Thoughts | 10.0 ±2.7 | 11.7 ±5.2 | 0.528 | 8.5 ±3.3 | 8.9 ±3.3 | 0.797 |
| - Cognitive self-consciousness | 11.6 ±4.2 | 10.8 ±3.1 | 1.000 | 8.8 ±2.0 | 9.8 ±3.3 | 0.433 |

Continuous variables are stated as mean and standard deviation (SD) and categorical variables are stated as n and percent (%), unless indicated otherwise. P-values were calculated using paired Wilcoxon signed-rank test. Statistically significant values in bold.
Abbreviations: WHO, world health organization; QoL, quality of life; HADS, hospital anxiety and depression scale; A, anxiety; D, depression; MCQ-30, Metacognitions questionnaire 30;

**Table S2 - QoL and psychometric scores in PAH patients before sotatercept compared to at least 6 months on sotatercept treatment by change of WHO FC**

|  | Not Improved WHO FC (n=12) | | | Improved WHO FC (n=8) | | |
| --- | --- | --- | --- | --- | --- | --- |
| Scores in points | **Before sotatercept** | **6 months on sotatercept** | **p value** | **Before sotatercept** | **6 months on sotatercept** | **p value** |
| WHOQOL-BREF |  |  |  |  |  |  |
| - Global | 52.1 ±19.8 | 50.0 ±16.5 | 0.746 | 56.2 ±11.6 | 64.1 ±22.6 | 0.586 |
| - Physical | 61.0 ±13.1 | 48.8 ±14.2 | **0.009** | 58.9 ±14.3 | 58.5 ±14.9 | 0.796 |
| - Mental | 69.4 ±22.4 | 44.8 ±17.5 | **0.008** | 69.8 ±16.8 | 53.1 ±13.7 | **0.039** |
| - Social | 59.7 ±20.0 | 63.9 ±21.7 | 0.905 | 67.7 ±23.3 | 54.2 ±12.6 | 0.093 |
| - Environmental | 75.5 ±14.8 | 74.0 ±15.0 | 0.512 | 81.6 ±10.8 | 77.7 ±13.1 | 0.395 |
| HADS |  |  |  |  |  |  |
| Total score | 10.3 ±8.7 | 10.2 ±7.3 | 0.475 | 9.9 ±8.3 | 11.1 ±6.1 | 0.865 |
| HADS-A Score | 5.1 ±4.8 | 4.9 ±3.9 | 0.878 | 5.2 ±5.0 | 6.2 ±3.4 | 0.798 |
| HADS-D Score | 5.2 ±5.2 | 5.3 ±4.2 | 0.384 | 4.6 ±3.7 | 4.9 ±4.2 | 1.000 |
| MCQ-30 |  |  |  |  |  |  |
| - Total score | 46.2 ±11.4 | 54.6 ±14.0 | 0.181 | 44.1 ±12.7 | 40.9 ±8.5 | 0.262 |
| - Positive beliefs about worry | 7.2 ±1.7 | 7.9 ±2.5 | 0.916 | 7.8 ±2.0 | 6.1 ±0.4 | 0.058 |
| - Cognitive confidence | 10.0 ±3.4 | 11.4 ±3.3 | 0.268 | 8.8 ±2.8 | 8.9 ±2.2 | 0.890 |
| - Negative beliefs | 9.1 ±3.5 | 7.9 ±2.5 | 0.141 | 9.9 ±4.9 | 9.0 ±3.5 | 0.498 |
| - Need to Control Thoughts | 9.6 ±3.5 | 11.8 ±4.7 | 0.232 | 8.4 ±2.3 | 7.8 ±2.1 | 0.461 |
| - Cognitive self-consciousness | 10.2 ±3.7 | 11.1 ±3.6 | 0.282 | 9.4 ±2.7 | 9.1 ±2.5 | 0.672 |

Continuous variables are stated as mean and standard deviation (SD) and categorical variables are stated as n and percent (%), unless indicated otherwise. P-values were calculated using paired Wilcoxon signed-rank test. Statistically significant values in bold.
Abbreviations: WHO, world health organization; QoL, quality of life; HADS, hospital anxiety and depression scale; A, anxiety; D, depression; MCQ-30, Metacognitions questionnaire 30;

**Table S3 – Sotatercept specific questions after at least 6 months on sotatercept treatment – n=17 patients participated**

| Question | Does not apply/never | Rarely | Sometimes | Often | Totally true/always |
| --- | --- | --- | --- | --- | --- |
| Before entering the study, I feared for my life | **6** | 3 | 4 | 3 | 2 |
| Currently I fear for my life | 5 | **8** | 2 | 2 | 0 |
| I feel relieved | 6 | 1 | 2 | **6** | 2 |
| Since I've been in this study, I have a more positive outlook on life/have more hope | 4 | 1 | 2 | **7** | 3 |
| I wouldn't have believed that my condition could improve so much | 7 | 1 | 2 | 3 | 4 |
| For the first time, I have hope for a normal life expectancy | 5 | 2 | 4 | **5** | 1 |
| I can finally do things I wanted to do for a long time | 4 | **5** | 3 | **3** | **2** |
| I am planning to travel again | 3 | 4 | 3 | 3 | **4** |
| I set myself more long-term goals than before the study | **7** | 4 | 2 | 2 | 2 |
| I have restarted a hobby or looked for other activities, for example volunteering, choir, evening school, sports | **8** | 3 | 1 | 2 | 3 |
| I also feel fitter mentally | 6 | 3 | **5** | 2 | 1 |
| I need to reorient/define myself personally | 7 | 3 | 3 | 1 | **3** |
| My illness has defined/dominated my purpose in life so far | 1 | 2 | 5 | **6** | 3 |
| I have no regular pension entitlement | **14** | 1 | 0 | 0 | 1 |
| I can start working again | **12** | 1 | 0 | 0 | 4 |
| I can work in my old job | **8** | 2 | 0 | 0 | 7 |
| I have to reorientate myself professionally | **16** | 1 | 0 | 0 | 0 |
| I can reorientate myself professionally | **12** | 2 | 1 | 1 | 1 |
| I fear for my social security benefits due to the health improvements that occurred in the study | **12** | 1 | 4 | 0 | 0 |
| My economic existence is at risk | **11** | 1 | 4 | 0 | 1 |
| The financial impact of my health improvement scares me | **11** | 3 | 3 | 0 | 0 |
| The relationship with those around me has changed | **8** | 4 | 2 | 1 | 2 |
| My relationship with my partner has changed | **11** | 3 | 0 | 1 | 2 |
| Since start of the study, the topic of having children has concerned me more than before | **16** | 0 | 0 | 0 | 1 |
| I feel guilty that other patients are doing worse without study medication | **12** | 4 | 1 | 0 | 0 |
| I feel guilty that other patients died | **13** | 3 | 1 | 0 | 0 |
| I feel guilty that other patients couldn't get into the study | **12** | 3 | 1 | 1 | 0 |
| Sometimes I don't dare to tell how well I'm feeling because other people are not | **10** | 2 | 2 | 2 | 1 |

Categorical variables are stated as n and percent (%), unless indicated otherwise. Translated question in bold, original Question in German language below. Values with highest occurrence are marked as bold.
